# Effects of Higher Dietary Protein Intake on Isokinetic Muscle Performance in Older Adults with Type 2 Diabetes Mellitus

**DOI:** 10.3390/jfmk11010125

**Published:** 2026-03-19

**Authors:** Stavroula Kalyva, Dionysia Argyropoulou, Panagiotis Koulouvaris, Charilaos Tsolakis, Gerasimos Terzis, Tzortzis Nomikos, Nickos D. Geladas, Anastasios A. Theodorou, Vassilis Paschalis

**Affiliations:** 1 School of Physical Education and Sport Science, National and Kapodistrian University of Athens, 17237 Athens, Greece; 2 Sports Excellence, 1st Orthopedics Department, School of Health Sciences, National and Kapodistrian University of Athens, 12462 Athens, Greece; info@drkoulouvaris.gr; 3 Department of Nutrition and Dietetics, Harokopio University, 17676 Athens, Greece; tnomikos@hua.gr; 4 Department of Life Sciences, School of Sciences, European University Cyprus, 2404 Nicosia, Cyprus; a.theodorou@euc.ac.cy

**Keywords:** elderly, isokinetic dynamometry, Mediterranean diet, muscle strength, sarcopenia

## Abstract

**Background**: Type 2 diabetes mellitus (T2DM) is linked to accelerated losses in muscle function. The aim of the present investigation was to evaluate the effect of chronic higher-protein intake on isokinetic knee performance in the older adults with type 2 diabetes mellitus (T2DM). **Methods**: Thirty adults (15 men and 15 women) aged 60–80 years with non-insulin-treated T2DM and sarcopenia-related deficits were randomized for 12 weeks to a higher-protein diet (1.2–1.5 g·kg^−1^·day^−1^) or a recommended protein diet (0.8–1.0 g·kg^−1^·day^−1^), with meal plans designed to maintain body mass. Protein was increased mainly through Mediterranean-style protein sources while diet was monitored using repeated 3-day recalls. Isokinetic knee extensors and flexors peak torque (angular velocity 60°/s) was assessed at baseline and at week 6 and at the end of the 12th week of intervention. Fatigability (20 maximal contractions at an angular velocity at 180°/s) and DXA body composition were assessed at baseline and at the end of the 12th week of intervention. Data were analyzed using two-way repeated-measures ANOVA with repeated measures on time (2 groups × 3 time points), followed by post hoc analysis when significant difference was occurred. **Results**: Thirty participants completed the trial. Energy intake and body mass remained stable in both groups. At the end of week 12, peak torque declined in the control group (*p* < 0.05) but remained stable in the higher-protein group. The fatigue index worsened over time in controls but was preserved in the higher protein group, resulting in a significant group d7 time interaction at week 12 (*p* < 0.05) (post hoc between-group difference at week 12, *p* < 0.05). Fat-free mass and blood pressure did not change. In fat mass, a decrease was observed with higher protein intake, whereas it remained stable in the control group. **Conclusions**: Higher protein intake for 12 weeks preserved knee muscle performance in older adults with T2DM without weight gain, supporting dietary protein optimization to counter functional decline.

## 1. Introduction

Type 2 diabetes mellitus (T2DM) is highly prevalent in older adults and is characterized by insulin resistance [1,2,3]. In skeletal muscle, impaired insulin signaling reduces GLUT4-mediated glucose uptake, contributing to hyperglycemia and compromised substrate availability for muscle metabolism [4]. Beyond its metabolic and vascular complications, T2DM is increasingly associated with accelerated losses in skeletal muscle mass and strength, thereby increasing the risk of sarcopenia, functional limitations, and loss of independence [5,6,7].

Sarcopenia is a progressive, generalized skeletal muscle disorder characterized by decline in muscle strength and function and is associated with fragility and increased mortality [8,9]. Evidence supports a bidirectional relationship between sarcopenia and T2DM, whereby sarcopenia may not only be a cause for diabetes but also a consequence [6,7,10]. Recent data show that decline in skeletal muscle mass and function is greater in patients with T2DM compared to age-matched individuals with a normal glycemic status [10,11,12,13,14,15]. Mechanistically, reduced insulin action may impair muscle protein synthesis and alter proteolysis, whereas muscle loss may further worsen glycemic control and inflammation, reinforcing this vicious cycle [16].

Adequate dietary protein intake, together with the anabolic effect of insulin, represents a modifiable factor for skeletal muscle mass maintenance in older adults with T2DM [17,18]. Protein provides essential amino acids for muscle protein synthesis [19] and has been linked to improved muscle quality and performance [20]. While general recommendations suggest ≈0.8 g/kg/day [21], older adults with acute or chronic disease may require up to ≈1.5 g/kg/day (or approximately 25–30 g/meal) to overcome anabolic resistance and stimulate postprandial muscle protein synthesis [22], while higher-protein dietary patterns are also supported by the 2025–2030 Dietary Guidelines for Americans [23]. Recent 12-week interventions in older adults with non-insulin-treated T2DM indicate that increasing protein intake to ≈1.2–1.5 g/kg/day preserves or improves functional performance compared with standard intake (≈0.8–1.0 g/kg/day) [12]. Specifically, higher protein intake has been associated with improvements in functional outcomes (e.g., timed-up-and-go, gait speed, standing balance) and handgrip strength, whereas standard intake has been accompanied by reductions in appendicular lean mass, skeletal muscle index, and handgrip strength [12]. Similarly, a 12-week trial reported preservation of appendicular lean mass and improvements in knee extensor strength under higher protein intake, with deterioration in appendicular lean mass under the lower-protein condition [11]. Notably, although women typically exhibit lower absolute performance than men, available evidence suggests comparable relative preservation/improvement after 12 weeks of higher protein intake between sexes [11,12]. Higher protein intake may also support glycemic regulation by enhancing insulin responses without increasing plasma glucose concentrations [24,25], and amino acids co-ingested carbohydrate can further stimulate insulin secretion, facilitating anabolism [26].

Most studies in older adults and T2DM populations have evaluated protein supplementation using static strength tests (e.g., handgrip) and global physical performance measurements (e.g., gait speed, sit-to-stand, Short Physical Performance Battery) [11,12,27,28]. However, these assessments may not fully capture dynamic muscle function and limited ability to quantify and standardize join-specific loading. Isokinetic dynamometry provides highly standardized assessment by maintaining constant angular velocity with controlled loading, providing objective indices (e.g., peak torque, endurance) with good reliability when standardized protocols are applied. Moreover, fixed positioning further supports consistent execution of the exercise and minimizes injury risk [29,30,31], while the same isokinetic dynamometer can also be used to assess muscle fatigability—an approach that is particularly advantageous in older adults because it enables repeated, voluntary maximal-effort contractions of small muscle groups (e.g., the knee extensors of one limb at an angular velocity 180°/s) under tightly standardized conditions [32]. Importantly, lower isokinetic knee extensor and flexor strength is associated with mobility limitation, increased fall risk, and disability in the older adults [33,34]. Nevertheless, the effects of longer-term dietary protein manipulation on isokinetic muscle function in older adults with T2DM remain insufficiently studied.

Therefore, the present investigation aimed to examine the effects of 12 weeks of higher-protein intake (1.2–1.5 g/kg/day) versus recommended intake (0.8–1.0 g/kg/day) on isokinetic knee muscle function in the older adults with non-insulin-treated T2DM. We hypothesized that the higher protein diet would preserve or improve isokinetic knee muscle strength compared with the control diet, without significant changes in body weight or energy intake.

## 2. Materials and Methods

### 2.1. Participants

Thirty-seven individuals were initial screened (i.e., 18 men and 19 women). Four subjects were excluded for not meeting the inclusion criteria. Of the remaining 33 participants, three were excluded from the final analysis (one from CG and two from IG) because they did not follow the prescribed diet or missed scheduled follow-up visits. Ultimately, 30 participants (i.e., 15 men and 15 women), aged 60–80 years, with non-insulin-dependent T2DM diagnosed within the previous 10 years (Table 1), were included in the analysis. All included participants also had either low muscle mass, low muscle strength, or low physical performance. Based on self-reported information, subjects were classified into low muscle mass or low muscle strength or low physical performance, similar to criteria for the diagnosis of sarcopenia from the European Working Group of Sarcopenia (EWGSOP2), which involves three main categories (differing for each sex): muscle mass, muscle strength and physical performance [35]. Hospital doctors have diagnosed T2DM in accordance with the American Diabetes Association [1]. That is, T2DM was diagnosed either based on plasma glucose criteria (≥200 mg/dL), or based on the 2 h plasma glucose value (≥200 mg/dL during 75 g oral glucose tolerance test) value, or on the fasting plasma glucose (≥126 mg/dL), or on A1C criteria (≥6.5%). All female participants were at menopause. Exclusion criteria included: (a) receiving dietary compliments or following another dietary intervention during the study or one month before the study, (b) having nephropathy or cancer, (c) cognitive impairments, (d) neurological disorders, (e) previous surgery that would deteriorate their motion during the assessments, and (g) body mass index (BMI) > 40.

All potential participants were informed about the objectives, methodology, and implications of the study and were required to sign a written consent form prior to entry. After consent was obtained, the interested participants were screened for eligibility. The overall project complied with clinical publications practices and was reviewed and approved by the Ethics Committee of the National and Kapodistrian University of Athens (Protocol Record 1288/03-07-2021), and was registered at ClinicalTrials.gov (NCT05457088) on 15 July 2022. The procedures were in accordance with the standards set by the latest version of the Helsinki Declaration (19 October 2013).

### 2.2. Study Design

Eligible participants were randomly allocated to the control group (CG) or to the intervention group (IG) in a 1:1 ratio using sex-stratified randomization to ensure equal representation of men and women across groups. Within each sex division, participants were assigned to IG or CG at a 1:1 ratio (15 participants per group overall; 7 men and 8 women in the CG and 8 men and 7 women in the IG). Participants were administered a diet plan in person (based on their weight and aimed in maintaining energy intake) with either the current recommended protein intake (CG; 0.8–1.0 g/kg/day) or a diet plan based on the dietary guidelines for Americans, that is 1.2–1.5 g/kg/day protein intake [23]. Individualized meal plans were developed by a dietitian based on participants’ body mass and estimated energy requirements, with the goal of maintaining stable body weight. The individualized meal plans were designed to cover all meals consumed across the day. Protein intake was increased primarily by incorporating multiple Mediterranean-style protein sources (lean meat, poultry, fish, eggs, dairy, and legumes) [36]. Fat intake was controlled by prescribing both the type and exact amount of fat, distributing it across meals, and ensuring the total daily energy intake matched the individualized target [1]. Carbohydrate intake followed usual dietary patterns compatible with T2DM nutritional guidelines.

Participants received in-person diet counseling at baseline and were provided with sample menus and portion guides. Adherence was reinforced during follow-up visits at week 6 and via periodic phone contacts. During this period, three study visits were scheduled: at the beginning of the trial (baseline), after 6 weeks of intervention and at the end of treatment (12 weeks). At baseline, in addition to the administered diet plan, participants had their anthropometric characteristics and body composition measured. They were also assessed for their isokinetic performance and nutritional habits. At the end of the intervention period (i.e., week 12), all of the above measurements were repeated. Follow-up measurements were performed at week 6 except for body composition assessed using DXA (Lunar Prodigy, General Electric Medical Systems, Madison, WI, USA) to minimize radiation exposure and the isokinetic fatigue test due to maximal intensity requirements. Throughout the 12-week intervention, participants were instructed not to change their usual lifestyle or physical activity pattern and to continue with their normal day-to-day activities, so that any observed changes could be attributed primarily to the dietary intervention.

### 2.3. Anthropometric Characteristics

A weight scale (model 700, SEGA, London, UK) with telescopic measuring rod for SEGA column scales (model 220, SEGA, London) was used to measure body mass and height, respectively. The body mass index (BMI) was subsequently calculated as the ratio between body mass (kg) and the square of height (m^2^). Systolic arterial pressure, diastolic arterial pressure and pulse were also measured using a common sphygmomanometer (SECA b12, London, UK). All the above measurements were assessed at weeks 0, 6, and 12. Body composition, muscle mass, fat mass and free fat mass were derived using dual-energy X-ray absorptiometry (Lunar Prodigy, General Electric Medical Systems, Madison, WI, USA). Body composition assessed by DXA shows good validity against criterion methods [37] and excellent test–retest reliability, with very high repeatability for total-body measures (ICC ≳ 0.99 and low CVs reported in reproducibility studies [38,39]. Participants had their body composition measured at weeks 0 and 12. All measurements were carried out by the same trained technician and the equipment was calibrated daily according to manufacturer specifications.

### 2.4. Dietary Analysis

Dietary intake was assessed using repeated 3-day dietary recalls [40] collected from all participants at baseline (week 0) and at weeks 6 and 12 of the intervention. All recalls were administered by the principal investigator and were used in the assessment of nutritional habits and the macronutrient analysis (i.e., protein, carbohydrates, lipids). Diet analysis was essential in order to check the adherence of the participants to the instructed diets. FoodData database (U.S Department of Agriculture) was used for the diet analysis [41].

### 2.5. Isokinetic Muscle Function Assessment

The participants had their isokinetic muscle strength measured at weeks 0, 6, and at the end of the 12th week of intervention. All isokinetic assessment were performed early in the morning between 8 and 10 AM. Participants were instructed to avoid participate in any physical demanding activity during the two days prior to isokinetic performance assessment. Procedures were fully explained before assessment followed by a familiarization attempt. The isokinetic assessment, consisting of concentric muscle contractions, was performed on an isokinetic dynamometer (Biodex System 4 PRO, Biodex Medical Systems Inc., NY, USA). The participants performed a familiarization isokinetic session for the knee extensors, which consisted of eight concentric contractions of very low intensity at 60°/s. After a 5 min rest period, participants performed five isokinetic concentric knee extensor repetitions at 60°/s were performed, of which the final three were maximal voluntary contractions (MVC), and the average of the two highest value was recorded for the statistical analysis. After a 10 min interval, participants performed the fatigue test, which consisted of 20 concentric maximally voluntary contractions at an angular velocity of 180°/s. The fatigue index was calculated as the percentage difference in torque by the following formula [42]: Fatigue index = 100 − (average torque of the 5 last repetitions/average torque of the 5 first repetitions) × 100. (this assessment was performed only in weeks 0 and 12). The range of motion during isokinetic dynamometry was 90° [knee range, 10° (full extension) to 100° flexion]. Isokinetic concentric peak torque was assessed for both limbs and the average value of the best performance for each limb was used for the statistical analyses. The dominant leg was identified as the preferred limb, while, at each assessment (i.e., weeks 0, 6, and at the end of the 12th week of intervention), the limb tested first was randomly selected. A 2 min rest period was allowed between legs. Verbally encouragement was provided to maximize effort. The participants were instructed to initiate each contraction from a fully relaxed state, and no intentional pre-tension was applied before the onset of the lever leg movement. The dynamometer began recording at the start of movement. The isokinetic dynamometer was calibrated according to the manufacturer’s guidelines. The participants’ trunk and pelvis were strapped to minimize compensatory movements, and the tested limb was secured to the lever arm proximal to the malleoli. The dynamometer rotational axis was aligned with the lateral femoral epicondyle. Gravitational corrections were applied to account for the passive torque produced by the limb’s weight when at rest. These corrections ensured that only active muscle-generated torque was analyzed during the contraction phases. Before the isokinetic assessment, participants performed an 8 min warm-up on a cycle ergometer (Monark 894E Peak Bike, Vansbro, Sweden) at 40 rpm and 25 W. This standardized warm-up ensured uniformity across participants, particularly those unfamiliar with laboratory tests.

### 2.6. Statistical Analysis

Shapiro–Wilk test was used to assess the normality of our data and no violation of normality was found (*p* > 0.05). An a priori sample size calculation was conducted using G*Power (version 3.1.9.6) for an F-test (ANOVA: fixed effects, special, main effects and interactions). Based on an effect size of 0.90 derived from our previous investigation of the fatigue index in older adults [32], with statistical power set at 85%, the required sample size was estimated accordingly. The sample size was estimated a priori to using G*Power (ver. 3.1.9.6) to achieve 85% statistical power for detecting an effect size of 0.90, based on our previous investigation of the fatigue index in older adults. Baseline differences between groups were examined using independent-samples t-tests. A two-way repeated-measures ANOVA [groups (control and intervention) × time (pre, 6, and 12 weeks)] was used to analyze dietary intake, anthropometrics, isokinetic performance and fatigue index. For DXA-derived outcomes, the time points were pre and 12 weeks only. A three-way repeated-measures ANOVA [groups (control and intervention) × time (pre and 12 weeks) × repetitions (1–5, 6–10, 11–15 and 16–20 repetitions)] was used to analyze isokinetic fatigue test. When a significant interaction was detected, pairwise comparisons were performed with Bonferroni correction to control for multiple testing (i.e., comparisons between groups at each time point and/or comparisons across time within each group; for the fatigue protocol, comparisons across repetition blocks within each time point and group were similarly adjusted). Statistical significance was set at *p* < 0.05. In addition to *p*-values, results are reported with effect sizes and 95% confidence intervals (CI) where appropriate. For repeated-measures ANOVA effects (main effects and interactions), partial eta squared (η_p_^2^) is reported. Data are presented as mean ± SD. All analyses were performed using IBM SPSS Statistics version 25.0 (IBM Corp., Armonk, NY, USA).

## 3. Results

### 3.1. Participants

The intervention did not affect systolic or diastolic blood pressure, body mass or free fat mass (Table 1). Regarding BMI, despite a significant main effect of time [F(2,27) = 3.6, *p* = 0.034, η_p_^2^ = 0.114], post hoc analysis did not reveal any significant difference over time in either group [CG Δ = 4.6, 95% CI [25.5, 30.1); IG Δ = 6, 95% CI [25.1, 30.1)]. Percent fat mass showed no main effect of group [F(1,28) = 0.008, *p* = 0.130, η_p_^2^ = 0.037]. However, there was main effect of time [F(1,28) = 8.99, *p* = 0.029, η_p_^2^ = 0.301] and a significant time × group interaction [F(1,28) = 11.6, *p* = 0.002, η_p_^2^ = 0.294]. In CG, percent fat mass differed from baseline to week 12 by Δ = −1.3 (95% baseline [30.8, 39.2] and week 12 [32.2, 40.4]). In the IG, percent fat mass differed from baseline to week 12 by Δ = 1.2 (95% baseline [29.1, 31.5] and week 12 [28, 36.2]).

### 3.2. Dietary Assessment

Participants in both groups successfully followed the scheduled dietary plans (Table 2). As designed, protein intake relative to body mass differed significantly between groups at weeks 6 (CG 0.83 ± 0.06 vs. IG 1.24 ± 0.15) and 12 (CG 0.83 ± 0.07 vs. IG 1.39 ± 0.08) [F(2,27) = 123, *p* < 0.001, η_p_^2^ = 0.814]. In the IG protein intake (g·kg^−1^·day^−1^) increased from baseline to weeks 6 and 12 (0.81 ± 0.13, 1.24 ± 0.15, 1.39 ± 0.08, respectively; *p* < 0.05) and remained higher than in the CG (*p* < 0.001). At week 6 and week 12, the CG differed from the IG by Δ = 0.41 (95% CG [0.77, 0.89] and IC [1.18, 1.3]) and Δ = 0.56 (95% CG [0.79, 0.87] and IC [1.35, 1.43]), respectively.

The increase in protein intake in the IG was accompanied by a reduction in lipid intake. Lipid intake differed significant between groups at week 12 [F(2,27) = 8.2, *p* = 0.001, η_p_^2^ = 0.227]. At week 12, the CG differed from the IG by Δ = 7.8 (95% CI [39.1, 45.6] and (95% CI [31.8, 37.8]), respectively. In the IG, lipid intake decreased versus baseline at weeks 6 and 12 (43.4 ± 10.6, 35.3 ± 8.8 and 34.5 ± 6.8, respectively; time × group interaction *p* = 0.001). Total energy intake did not differ over time or between groups (main effect of time: F(2,27) = 0.006, *p* = 0.994, η_p_^2^ < 0.001; main effect of group: F(2,27) = 0.006, *p* = 0.332, η_p_^2^ = 0.034). Although a significant time × group interaction was observed in total energy intake [F(2,27) = 6.1, *p* = 0.027, η_p_^2^ = 0.199)], post hoc analyses did not identify significant pairwise comparisons.

### 3.3. Isokinetic Performance of Knee Muscles

**Figure 1:**
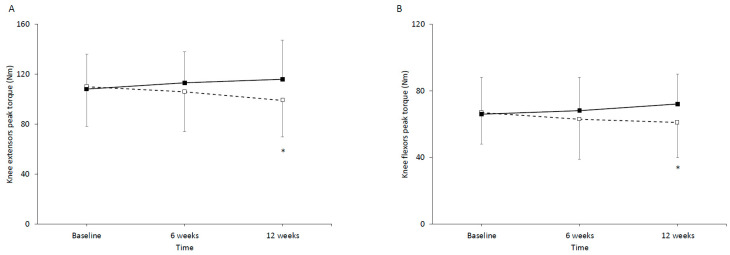
(**A**) Knee extensors and (**B**) knee flexors peak torque for the control (dashed line/white boxes) and the intervention (solid line/black boxes) group at baseline and at 6 and 12 week of the intervention. * Significant difference compared to the baseline for the same group.

Isokinetic knee extensor and flexor performance is shown in Figure 1. Knee extensor performance did not reveal main effect of time [F(2,27) = 0.784, *p* = 0.462, η_p_^2^ = 0.027] or main effect of group [F(2,27) = 0.478, *p* = 0.495, η_p_^2^ = 0.014]. However, a significant time × group interaction was observed [F(2,27) = 8.22, *p* < 0.001, η_p_^2^ = 0.227]. At the end of the 12th week of intervention, knee extensor performance decreased significant in the CG (*p* < 0.05), whereas no significant changes were observed in IG (*p* > 0.05). In the CG, baseline differed from week 12 by Δ = 10.3 (95% baseline [93.6, 125.5] and week 12 [83.3, 115.2]).

Knee flexor performance did not reveal main effect of time [F(2,27) = 0.162, *p* = 0.851, η_p_^2^ = 0.006] or main effect of group [F(2,27) = 0.476, *p* = 0.496, η_p_^2^ = 0.017]. However, there was time × group interaction [F(2,27) = 8.64, *p* = 0.001, η_p_^2^ = 0.236]. At the end of the 12th week of intervention, knee flexor performance decreased significant in the CG (*p* < 0.05), whereas no significant changes were observed in IG (*p* > 0.05). In the CG, baseline differed from 12 week by Δ = 6.2 (95% baseline [56, 77.6] and week 12 [50.2, 71]).

### 3.4. Isokinetic Fatigue Test and Fatigue Index

**Figure 2:**
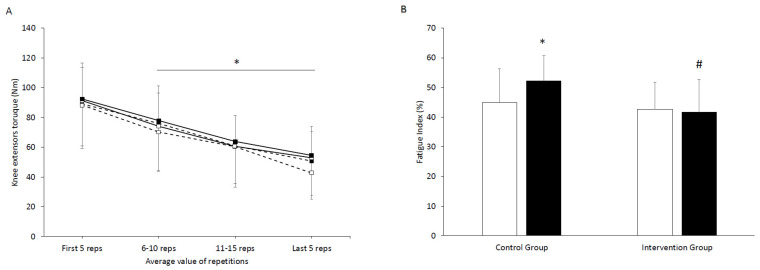
(**A**) Knee extensors torque for the control (dashed line) and the intervention (solid line) group during 20 repetitions of maximal voluntary intensity at baseline (black boxes) and at week 12 of intervention (white boxes). (**B**) Fatigue index for the control and the intervention group at baseline (white columns) and at week 12 of the intervention (black columns). * Significant difference compared to baseline (*p* < 0.05). # Significant difference between the two groups at the same time point (*p* < 0.05).

Isokinetic fatigue test results are shown in Figure 2A. Isokinetic fatigue test analysis showed main effect of repetition block [F(3,54) = 351, *p* < 0.001, η_p_^2^ = 0.862], indicating that torque declined progressively across repetitions. There was no main effect of group [F(3,54) = 0.372, *p* = 0.544, η_p_^2^ = 0.007] or main effect of time [F(3,54) = 0.013, *p* = 0.909, η_p_^2^ < 0.001]. There was also no significant repetitions by group interaction [F(3,54) = 1.58, *p* = 0.196, η_p_^2^ = 0.027] or repetitions by time interaction [F(3,54) = 1.05, *p* = 0.374, η_p_^2^ = 0.018] or group by time interaction [F(3,54) = 0.272, *p* = 0.374, η_p_^2^ = 0.005] or repetitions by group by time interaction [F(3,54) = 1.12, *p* = 0.334, η_p_^2^ = 0.02]. During the isokinetic fatigue test (Figure 2A), torque declined progressively to the 20th repetition in both groups (*p* < 0.05). No between-group differences in fatigue test scores were observed at week 6 or at the end of the 12th week of intervention. Nevertheless, based on post hoc comparisons, the CG showed a tendency towards a greater decline during the final five repetitions of week 12 (main effect of time *p* = 0.082). At the end of the 12th week of intervention, isokinetic fatigue test in CG differed from the IG by Δ = −11.7 (95% CG [32.7, −52.9] and IG [44.3, 64.6]).

Fatigue index results are shown in Figure 2B. The fatigue index showed a main effect of group [F(1,28) = 5.02, *p* = 0.033, η_p_^2^ = 0.152], but no main effect of time [F(1,28) = 2.14, *p* = 0.155, η_p_^2^ = 0.071] and no time × group interaction [F(2,27) = 3.27, *p* = 0.081, η_p_^2^ = 0.105]. At the end of the 12th week of intervention, fatigue index changed significant in the CG (baseline vs. week 12 Δ = −7.5 (95% baseline [−50.3, −39.3] and week 12 [−57.5, −47.2]). At the end of 12th week, the CG differed from the IG by Δ = 10.6 (95% CG [−57.5, −47.2] and IC [−46.9, −36.6]).

## 4. Discussion

The present randomized dietary intervention investigated whether increasing daily protein intake to 1.2–1.5 g·kg^−1^·day^−1^ for 12 weeks could preserve isokinetic knee muscle function in older adults with non-insulin-treated T2DM. The main finding was that, at the 12th week of intervention, isokinetic knee extensor and flexor performance declined in the CG consuming 0.8–1.0 g·kg^−1^·day^−1^, whereas performance was maintained in the IG consuming 1.2–1.5 g·kg^−1^·day^−1^. These functional changes occurred without meaningful differences in total energy intake, body mass, free fat mass, or blood pressure, indicating that the intervention primarily reflected macronutrient redistribution rather than an energy-restricted diet.

### 4.1. Diet Intervention and Muscle Function

Older adults with T2DM are at heightened risk for accelerated decline in muscle mass and strength, contributing to functional limitations, increased sarcopenia risk, and loss of independence [5,6,7]. This vulnerability is mechanistically plausible given impaired insulin signaling, reduced GLUT4-mediated glucose uptake and compromises substrate availability for skeletal muscle metabolism [43]. In addition, evidence supports a bidirectional relationship between sarcopenia and T2DM, whereby muscle loss can worsen glycemic control and inflammation, while diabetic metabolic dysfunction can accelerate muscle deterioration [6,7,10,16]. Against this background, the decline observed in the CG is consistent with the expected trajectory of progressive functional impairment in this population. In contrast, the maintained isokinetic performance in the IG supports the concept that dietary protein is a key modifiable determinant of muscle function preservation in older adults with T2DM [17,18]. Protein provides essential amino acids required for muscle protein synthesis [19] and has been associated with improved muscle quality and performance [20]. Given anabolic resistance in older adults and those with chronic diseases, higher protein intakes (≈1.5 g·kg^−1^·day^−1^ or ≈25–30 g per meal) may be required to maximize postprandial muscle protein synthesis responses [22,23]. The present results align with the rationale that higher-protein intake was sufficient to counterbalance functional decline over 12 weeks, even without measurable increases in fat-free mass.

Previous studies have reported heterogeneous findings regarding the effects of protein supplementation on muscle strength in older adults [44,45]. Differences in baseline nutritional status, type and amount of protein, presence of exercise training, and outcome measures may explain these discrepancies. Notably, many interventions used fixed absolute protein doses rather than body-mass-adjusted intakes, potentially leading to underdosing in heavier or more catabolic individuals. By targeting 1.2–1.5 g/kg/day, our protocol ensured that participants with higher body mass received proportionally higher protein intake.

A key aspect of the present findings is that isokinetic performance declined in the absence of detectable changes in free fat mass. Specifically, the CG showed a decline in knee extensor and flexor performance and the IC maintained performance, while in both groups, fat-free mass was stable. This pattern is compatible with the interpretation that higher protein intake may preferentially preserve muscle quality and neuromuscular function (i.e., force production relative to muscle size) rather than induce hypertrophy in the absence of a structured exercise stimulus. In practical terms, meaningful hypertrophy in older adults is most consistently achieved when adequate protein intake is combined with progressive resistance training, that is, in the absence of structured resistance training, increased protein intake may support net protein balance and reduce catabolic loss, but may be insufficient to stimulate measurable accretion of muscle tissue over a 12-week period. This is the case in T2DM, where impaired insulin action may blunt protein synthesis and alter proteolysis [16], and insulin resistance and impaired glucose handling can compromise muscle energetics and contractile function [1]. Increasing amino acid availability may therefore help sustain contractile performance even when whole-body lean mass changes are small and difficult to detect.

In parallel, the divergent fat–mass responses between groups may also be relevant. Percent fat mass increased in the control group, whereas fat mass decreased in the intervention group. Although total energy intake did not differ, this shift may reflect improved substrate partitioning with the higher-protein prescription, which can support metabolic regulation through improved insulin responses without raising plasma glucose [24,25]. Moreover, amino acids in combination with glucose can stimulate insulin secretion, potentially facilitating anabolism and supporting muscle maintenance [26]. Given the tight link between adiposity, inflammation, and muscle dysfunction in T2DM-related sarcopenia [10,16], even modest reductions in fat mass may contribute indirectly to preserved muscle function.

### 4.2. Isokinetic Assessment

Most previous investigations in older adults with T2DM have relied on handgrip strength and global functional tests (e.g., gait speed, sit-to-stand, Short Physical Performance Battery) [11,12,27,28]. While clinically useful, these outcomes may not fully capture dynamic joint-specific, velocity controlled muscle function or provide standardized loading across individuals. In contrast, isokinetic dynamometry allows controlled angular velocity, standardized positioning, and objective outputs (e.g., peak torque and fatigue indices), improving measurement precision when protocols are standardized [29,30,31]. This methodological advantage is important when interpreting the present findings. The observed between-group divergence in knee extensor and flexor performance at week 12 suggests that isokinetic testing may detect early diet-related changes in dynamic lower-limb function that are not reflected in whole-body lean mass measures. This is also clinically relevant because knee extensor and flexor performance is strongly linked to mobility limitations, fall risk, and disability in older adults [33,34]. Moreover, dietary restoration of deficiencies seems to have positive effects in performance outcomes in older individuals [32]. Therefore, the maintenance of knee extensor and flexor performance in the IG may have meaningful implications for mobility preservation in older adults with T2DM, even in the absence of detectable hypertrophy.

During the fatigue protocol, torque declined progressively across repetitions in both groups, consistent with the expected neuromuscular and metabolic demands of repeated maximal contractions. Although the torque-repetition profile did not differ significant between-groups at weeks 6 and 12, the CG showed a trend toward greater decline in the final five repetitions at week 12. In parallel, the fatigue index worsened in the CG at the end of 12th week of intervention while remained stable in the IG, with a significant between-group difference at week 12. Overall, these results suggest that the higher-protein diet may have been more effective for maintaining maximal isokinetic performance (strength/peak torque) than for inducing large changes in fatigability within 12 weeks. Given the complex determinants of fatigue (metabolic control, muscle oxidative capacity, and neuromuscular factors), the fatigue test may require either a longer intervention duration, larger sample size, or combined approaches (e.g., resistance training in addition to higher protein intake) to elicit clear between-group divergence.

### 4.3. Limitations and Practical Implications

The findings should be interpreted within the study’s limitations. The modest sample size may have limited statistical power to detect smaller between-group effects, particularly for fatigue-related outcomes. Physical activity was not objectively monitored (e.g., accelerometry) or quantified using a validated questionnaire during the intervention. Although participants were instructed to maintain their usual lifestyle, unrecorded changes in physical activity could have influenced energy balance and muscle performance outcomes. In addition, given the dietary implications of increased protein intake, it should be clarified that participants with known kidney disease were not included in the study. Finally, while higher protein intake is advocated for older adults with chronic conditions [22], the intervention duration may also have been insufficient to capture longer-term changes in muscle mass and neuromuscular function fully.

In the present investigation, diet assessed using repeated dietary recalls. Although this approach is practical and commonly used, it has limitations that are particularly relevant in older adults. Recall-based methods are vulnerable to memory-related error, underreporting (especially of snacks and portion sizes), social desirability bias, and high day-to-day variability, which may attenuate true between-group differences and reduce the precision of estimated macronutrient intake. In future trials, dietary exposure assessment could be strengthened by combining recalls with (i) multiple-day weighed food records in a subsample; (ii) photo-assisted food logging or digital tracking tools to improve portion-size estimation; (iii) periodic unannounced recalls to reduce anticipatory reporting; and (iv) objective biomarkers where feasible (e.g., urinary nitrogen as a supportive indicator of protein intake), alongside repeated counseling to standardize reporting quality. These enhancements would increase confidence that observed functional outcomes track true differences in dietary protein intake rather than measurement error.

Another limitation is that hydration status, including bioelectrical variables such as phase angle, was not evaluated or reported. As a result, the present findings cannot determine whether the observed body composition profile was accompanied by normal or improved hydration-related cellular health. Future studies should incorporate such measures to provide a more comprehensive interpretation of body composition changes and nutritional status.

The women participants in our study were in menopause using menopausal hormone therapy; however, current evidence in adults with T2DM does not support robust, consistent sex differences in glycemic responses to structured exercise interventions overall [46]. Importantly, our study was not powered to test sex-by-training/diet interactions; therefore, potential modification of intervention responsiveness by menopausal stage and/or menopausal hormone therapy cannot be excluded and should be addressed in future trials with prespecified subgroup analyses.

Despite these limitations, the present study supports the clinical rationale that higher protein intake may help counter functional decline in older adults with T2DM [6,7,10]. By preserving isokinetic knee strength and resistance to fatigue without altering total energy intake or body mass, the dietary strategy used here may represent a feasible nutritional approach for maintaining lower-limb function, which is central to mobility and independence [33,34]. Importantly, the intervention relied on dietary modification rather than commercial supplementation, potentially providing a broader amino acids profile, including essential amino acids, which may support strength and physical performance in the older adults and individuals with impaired insulin action [5]. It is plausible that the anabolic effects of insulin on muscle mass, together with adequate intake of high-quality proteins (i.e., essential amino acids), may represent an effective countermeasure for muscle loss [15,47]. Additional strengths of this study include objective assessments of muscle function using standardized isokinetic protocols and body composition assessed by dual-energy X-ray absorptiometry (DXA), which provides whole-body estimates of fat mass and fat-free mass.

### 4.4. Practical Implications and Future Guidelines

From a clinical standpoint, improvements/preservation of isokinetic knee muscle strength is relevant, because lower-limb strength is a key determinant of gait, chair-rise performance, and balance, while people with T2DM are the increased risk of falls and fractures. Our findings suggest that nutritional optimization alone, even in the absence of structured training, can have measurable effects on dynamic muscle function over 12 weeks. Future trials should evaluate whether higher protein intake combined with resistance training produces additive or synergistic improvements in isokinetic strength, fatigability and muscle mass in older adults with T2DM. Despite the substantial genetic contribution to T2DM risk [48], evidence from gene–lifestyle research supports that behavioral factors can modify genetic susceptibility, specifically indicating that higher physical activity levels may attenuate the risk of impaired glucose regulation and T2DM associated with some genetic variants [49,50]. Moreover, resistance exercise is an established component of T2DM management, and meta-analytic evidence indicates that it improves glycemic control (e.g., lowers HbA1c) on average, although individual responses vary, potentially reflecting differences in baseline phenotype, among other factors [51]. Finally, extending intervention duration and tracking clinically meaningful outcomes (falls, gait parameters, sit-to-stand power) would further strengthen translational relevance.

## 5. Conclusions

In older adults with non-insulin-treated T2DM and sarcopenia-related deficits, 12 weeks of higher protein intake, as currently suggested by the dietary guidance for Americans (1.2–1.5 g·kg^−1^·day^−1^) [23], preserved isokinetic knee extensor and flexor performance (i.e., peak torque and resistance to fatigue), whereas consumption of 0.8–1.0 g·kg^−1^·day^−1^ was associated with decline. These functional changes occurred without significant alterations in body weight or fat-free mass, supporting the interpretation that higher protein intake may preserve muscle quality and neuromuscular performance in the absence of a resistance-training stimulus. The findings reinforce higher protein intake as a modifiable factor to support muscle function in T2DM-related sarcopenia [17,18,22] and highlight isokinetic dynamometry as a sensitive outcome for detecting diet-related changes in dynamic lower-limb function [29,30,31]. Larger, longer-term trials combining nutritional optimization with targeted resistance-training interventions are warranted to confirm these findings and determine their impact on clinical outcomes such as falls, disability, and quality of life.

## Figures and Tables

**Table 1:** Anthropometric and health-related parameters of the participants (n = 30).

	Group	Baseline	Week 6	Week 12	*P* *_t-test_*	*P*_time_	*P*_group_	*P*_time × group_
Sex (men%)	CG	53						
	IG	53						
Age (years)	CG	73 ± 5			0.842			
	IG	72 ± 7						
SAP (mmHg)	CG	142 ± 9			0.544			
	IG	140 ± 8						
DAP (mmHg)	CG	85 ± 7						
	IG	82 ± 8			0.418			
Height (m)	CG	1.73 ± 0.12			0.497			
	IG	1.70 ± 0.10						
Body mass (Kg)	CG	82.8 ± 16.3	82.6 ± 15.4	82.4 ± 14.6		0.074	0.697	0.311
	IG	81.1 ± 15.9	80.4 ± 15.2	79.7 ± 14.7				
BMI	CG	28.8 ± 4.6	28.8 ± 4.4	28.7 ± 4.2		**0.034**	0.291	0.893
	IG	28.1 ± 3.4	27.8 ± 5.2	27.6 ± 5.2				
Fat mass (%)	CG	35.0 ± 9.2		36.3 ± 9.0 *		0.929	0.308	**0.022**
	IG	33.3 ± 6.6		32.1 ± 6.1				
Fat mass (Kg)	CG	30.0 ± 12.9		30.7 ± 12.2		0.303	0.378	**0.006**
	IG	27.5 ± 12.6		26.1 ± 12.8 *				
Fat-free mass (Kg)	CG	52.9 ± 7.5		51.7 ± 7.4		0.145	0.653	0.069
	IG	53.5 ± 8.8		53.7 ± 8.2				

BMI: body mass index; CG: control group; DAP: diastolic arterial pressure; IG: intervention group; SAP: systolic arterial pressure; * significant difference compared to baseline in the same group.

**Table 2:** Diet analysis of macronutrients between groups at baseline, after 6 weeks of intervention and after 12 weeks of intervention.

	Group	Baseline	Week 6	Week 12	*P* *_t-test_*	*P*_time_	*P*_group_	*P*_time × group_
Protein (g/Kg/d)	CG	0.84 ± 0.06	0.83 ± 0.06	0.83± 0.07		**<0.001**	**<0.001**	**<0.001**
	IG	0.81 ± 0.13	1.24 ± 0.15 *^#^	1.39 ± 0.08 *^#^				
Proteins (%)	CG	12.4 ± 4.1	13.5 ± 2.5	13.9 ± 2.8		**<0.001**	**0.005**	**<0.001**
	IG	12.1 ± 3.3	15.7 ± 2.9 *^#^	18.9 ± 2.9 *^#^				
Lipids (%)	CG	41.9 ± 7.4	41.1 ± 8.8	42.3 ± 5.5		**0.029**	0.130	**0.002**
	IG	43.4 ± 10.6	35.3 ± 8.7 *	34.5 ± 6.8 *^#^				
Carbohydrates (%)	CG	45.7 ± 8.5	45.4 ± 14.8	43.8 ± 9.5		0.194	0.510	0.154
	IG	44.5 ± 10.6	49.1 ± 8.3	46.6 ± 14.9				
Energy (Kcal)	CG	2078 ± 532	1885 ± 461	1919 ± 429		0.994	0.332	**0.027**
	IG	2008 ± 567	2212 ± 468	2167 ± 520				

CG: control group; IG: intervention group; * significant difference compared to baseline in the same group; # significant difference between the two groups at the same time point.

## Data Availability

The data presented in this study are available on request from the corresponding author.
